# “Transcranial Direct Current Stimulation (tDCS) in managing pain and recovery: A clinical case of radial capitellum fracture”

**DOI:** 10.1016/j.ijscr.2023.109120

**Published:** 2023-12-06

**Authors:** Roberto Tedeschi, Lisa Berti, Daniela Platano

**Affiliations:** a Department of Biomedical and Neuromotor Sciences, Alma Mater Studiorum, University of Bologna, Bologna, Italy (DIBINEM); b Physical Medicine and Rehabilitation Unit, IRCCS Istituto Ortopedico Rizzoli, 40136 Bologna, Italy

**Keywords:** Transcranial Direct Current Stimulation (tDCS), Radial capitellum fracture, Pain management, Physical rehabilitation, Non-pharmacological interventions

## Abstract

**Introduction:**

The management of pain and functional recovery following a radial capitellum fracture poses a significant clinical challenge, especially in individuals whose professions, such as physiotherapy, demand optimal joint functionality. Transcranial Direct Current Stimulation (tDCS) emerges as a potential non-pharmacological intervention for pain management, necessitating exploration in the context of orthopedic injuries.

**Case presentation:**

A 41-year-old male physiotherapist presented with a MASON 2 radial capitellum fracture following a fall, experiencing notable pain (NPRS 6/7) and functional impairment (DASH 45/100, PRTEE 43/100). Conservative management, involving immobilization and potential surgical consideration, was employed, followed by tDCS for pain management. Post-tDCS, significant improvements were observed in pain and functional scores (NPRS to 0, DASH to 14.2, PRTEE to 7), alongside enhancements in range of motion and muscle strength.

**Clinical discussion:**

The application of tDCS showcased notable efficacy in pain reduction and functional improvement, highlighting its potential in augmenting pain management strategies post-fracture. However, the variability in responses and lack of standardized application protocols necessitate further research to optimize its clinical utility. The balance between immobilization for fracture healing and mobilization for preventing stiffness and facilitating recovery was pivotal in managing the fracture and ensuring functional improvement.

**Conclusions:**

This case underscores the potential of tDCS in managing pain and facilitating functional recovery in radial capitellum fractures, warranting further exploration and standardization of its application in clinical practice. The integrated, patient-centric approach, involving interdisciplinary collaboration and personalized care, was crucial in ensuring positive outcomes and provides a framework for managing similar orthopedic cases.

## Highlights

• tDCS significantly reduces elbow pain.
• Functional recovery enhanced by tDCS.
• Pain-free status achieved in two weeks.
• DASH and PRTEE scores greatly improved.
• Complete elbow motion restored post-tDCS.

## 1 Introduction

Managing acute pain, especially in post-traumatic contexts like bone fractures, poses a significant clinical challenge that impacts not only the patient's quality of life but also the timing and efficacy of rehabilitation [1]. Radial head fracture, a common injury of the elbow joint [2], is often associated with acute and chronic pain, functional limitation [3], and the potential development of long-term complications such as osteoarthritis [4].

Transcranial Direct Current Stimulation (tDCS) [5] emerges as a non-invasive neuromodulation technique, which has demonstrated potential in mitigating pain and enhancing rehabilitative pathways in various clinical conditions [6]. tDCS operates by modulating neuronal activity through the application of low-intensity electrical current to the scalp, thereby influencing the activity of the underlying brain areas and potentially modulating neural networks involved in pain perception [7].

This case report explores the application of tDCS in a patient experiencing acute pain following a radial head fracture, with the aim of investigating the efficacy and safety of this technique in modulating pain and facilitating the rehabilitative journey. The radial head fracture, characterized by a break in the joint at the head of the radius, can result from direct traumas and often requires a multidisciplinary approach to ensure optimal recovery [8].

In a clinical context where pain represents a significant barrier to rehabilitation and can compromise functional outcomes, the use of tDCS might offer an innovative, non-pharmacological approach to pain management [9]. This report aims to explore and discuss the applicability, benefits, and potential limitations of using tDCS in treating acute pain associated with radial head fractures, providing insights for future research and clinical applications in this field.

## 2 Case presentations

A 41-year-old white male physiotherapist independently arrived at the emergency department of an orthopedic institute, conscious and cooperative, after accidentally falling from a ladder. He complained of severe pain and immobility in his left elbow but no other injuries. The physical examination showed significant tension (Numeric Pain Rating Scale - NPRS 6/7) with no visible bruising. The elbow's active and passive mobility was limited in all directions, yet it remained stable. Radiographs confirmed a lateral fracture of the MASON 2 radial capitellum. Given his profession, a CT scan was planned to evaluate the necessity for surgery. This scan revealed a closed fracture of the radial capitulum. Following the CT, the patient had restricted flexion-extension (25–110°) with NPRS 6 pain, but free and painless pronation-supination. He was equipped with a thermoplastic elbow brace for one week, advised to mobilize his shoulder and wrist twice a day, and to use pain medication as needed.

**Fig. 1:**
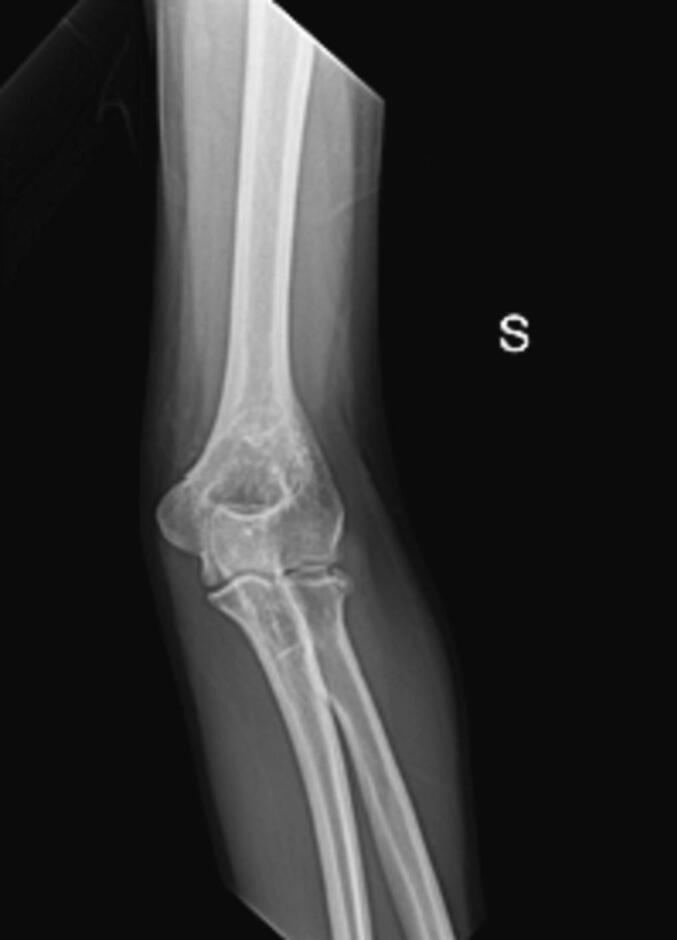
X-ray elbow.

**Fig. 2:**
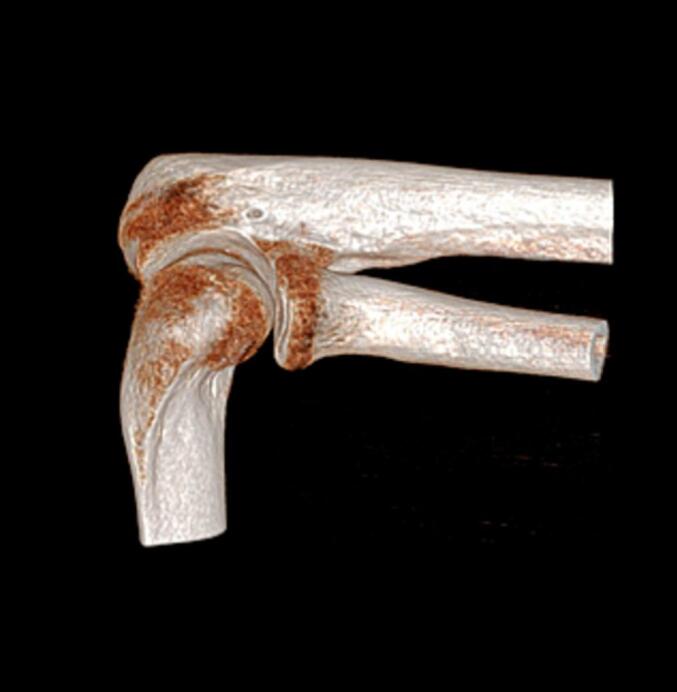
TC elbow.

**Fig. 3:**
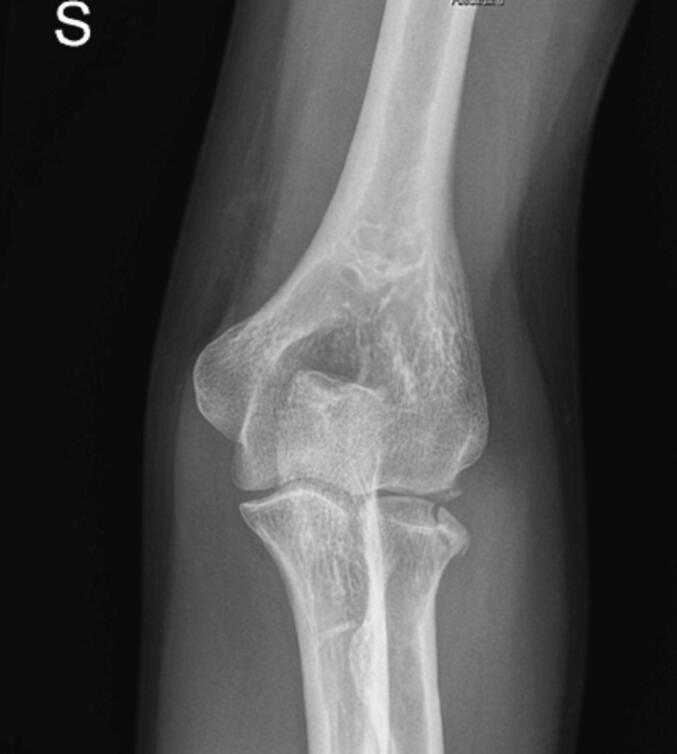
Xray elbow after 1 month.

A month after the injury, an X-ray affirmed the stability of the fracture. The elbow demonstrated improved movement in pronation-supination and flexion-extension (16–130°) with NPRS 4 pain. The patient underwent transcranial Direct Current Stimulation (tDCS) for two weeks for pain management. Initial evaluations indicated a DASH score of 45/100, a Patient-Rated Tennis Elbow Evaluation (PRTEE) score of 43/100, and an NPRS pain score of 4/10. The active range of motion (ROM) was: flexion 130°, extension 16°, pronation 175°, and supination 180°. Rotator cuff tests (Moving valgus stress test, Tinel Test) were negative. The Medical Research Council (MRC) scale rated muscle strength at 4/5 for both the biceps and triceps brachii, showing a moderate reduction in strength. After two weeks of tDCS (10 sessions), there were notable improvements: the DASH score reduced to 14.2, the PRTEE score to 7, and the NPRS pain score to 0. Upon re-evaluation, muscle strength remained stable at 4/5, and there was an improvement in the elbow's ROM (extension from 5° to 145°, complete pronosupination). The patient had a history of a surgically treated right clavicle fracture in 2016 and was not on any medication or known to have any allergies. This case study adheres to the SCARE [10](Surgical Case Report) guidelines for reporting surgical case studies. The SCARE guidelines aim to enhance the transparency and completeness of reporting surgical cases, providing a structured framework that facilitates accurate communication and assessment of surgical experiences (Fig. 1, Fig. 2, Fig. 3).

## 3 Clinical findings

### 3.1 Initial presentation and management

A 41-year-old male physiotherapist presented to the emergency department of an orthopedic institute following an accidental fall from a ladder. He experienced severe tension in his left elbow, rated 6/7 on the Numeric Pain Rating Scale (NPRS), with limited mobility but stable elbow. Radiographs confirmed a lateral fracture of the MASON 2 radial capitellum. Immediate management included pain control with analgesics and immobilization using a thermoplastic elbow brace. The patient was advised to mobilize his shoulder and wrist to prevent stiffness.

### 3.2 Follow-up and ongoing treatment

Post-CT scan, the patient showed limited flexion-extension (25–110°) with NPRS 6 pain, but free and painless pronation-supination. One month post-injury, his pain reduced to NPRS 4, with improved flexion-extension (16–130°) and free prone-supination. Radiographs confirmed fracture stability. For pain management, he underwent Transcranial Direct Current Stimulation (tDCS) for two weeks. Pre-tDCS assessments included a DASH score of 45/100, a PRTEE score of 43/100, and NPRS 4/10, with muscle strength rated at 4/5.

### 3.3 Post tDCS treatment outcomes

Two weeks post-tDCS, the patient showed remarkable improvement: DASH score reduced to 14.2, PRTEE to 7, and NPRS to 0. Muscle strength remained at 4/5, and ROM improved significantly. The therapeutic intervention included advanced physiotherapy and functional therapy, focusing on enhancing strength, stability, and functional use of the elbow.

### 3.4 tDCS in pain management

**Fig. 4:**
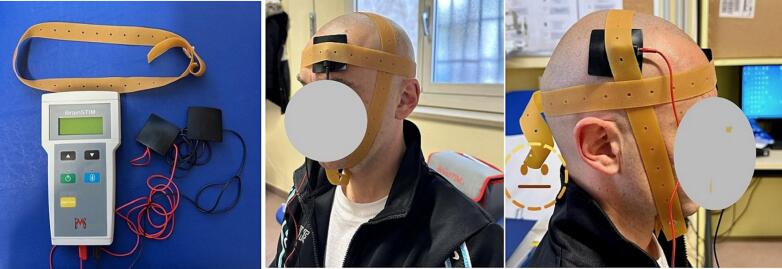
Transcranial Direct Current Stimulation (TDCS).

tDCS (Fig. 4), a non-invasive neuromodulation technique, was applied to manage pain and facilitate motor recovery. The patient underwent 10 sessions over two weeks, with electrodes placed over the motor cortex and pain matrix. The current was set at 1–2 mA for 20 min per session, following a standardized protocol for pain reduction. The procedure was monitored by a physiatrist and a physiotherapist to minimize measurement errors. No other parameters were considered in this treatment approach. The patient only took analgesics, paracetamol as needed. No anti-inflammatory treatment was necessary although it was prescribed.

### 3.5 Safety and efficacy

Transcranial Direct Current Stimulation (tDCS) is a non-invasive neuromodulation technique that has shown potential in pain treatment and enhancing motor functionality in various clinical contexts. In the presented case, tDCS was employed to manage pain and facilitate functional recovery in a patient with a radial capitellum fracture.

The side effects of tDCS are generally mild and transient. The most common include cutaneous sensations such as tingling, itching, or a slight burning sensation under the electrodes. Some patients may experience mild headaches or a feeling of fatigue or drowsiness post-treatment. Rarely, skin irritation may occur, especially with prolonged sessions or high frequency of treatment.

tDCS is considered safe for a wide range of patients, including adults and the elderly. However, there are some contraindications. Patients with neurological disorders like epilepsy might not be ideal candidates for tDCS. Additionally, the presence of metal implants in the head or implanted electronic devices (such as pacemakers) can represent a contraindication. The use of tDCS during pregnancy is not well studied, so caution is advised, as well as in the case of children and adolescents.

## 4 Discussion

The management of acute pain and the facilitation of functional recovery following a radial capitellum fracture, particularly in a patient whose profession in physiotherapy demands optimal elbow functionality, presents a nuanced and multifaceted challenge. The utilization of transcranial Direct Current Stimulation (tDCS) in this context not only underscores an innovative approach to managing pain but also highlights the potential of non-pharmacological interventions in modulating neuronal activity, especially within the motor cortex and pain matrix, to influence pain perception and motor recovery [11]. The notable improvement in pain levels and functional scores post-tDCS intervention, from an NPRS of 6 to 0 and DASH from 45/100 to 14.2, respectively, underscores its potential efficacy and provides a compelling avenue for further research and application in clinical settings. However, the variability in individual responses to tDCS, alongside considerations regarding optimal dosage and electrode placement, necessitates further research and the development of standardized guidelines for its application in diverse clinical scenarios. The conservative management of the MASON 2 radial capitellum fracture, involving a delicate balance between immobilization for fracture stability and early mobilization to prevent stiffness and facilitate recovery, was pivotal in ensuring stability and facilitating healing, as evidenced by the stability of the fracture at the one-month follow-up and the gradual improvement in ROM and functional scores. The patient's active engagement and feedback during the management, especially during tDCS sessions, enhanced the safety and efficacy of the interventions, highlighting the importance of a patient-centric approach in healthcare. Furthermore, the collaboration between orthopedic specialists, pain management teams, and rehabilitation professionals was crucial in providing comprehensive care, underscoring the importance of interdisciplinary collaboration in managing complex cases. This case provides valuable insights into the potential of tDCS in managing pain in orthopedic cases, warranting further research to explore its efficacy, mechanisms, and optimal application, and highlights the need for developing standardized guidelines for the application of tDCS in pain management, considering various pain conditions and individual variability. The long-term impact on elbow functionality, especially in the context of the patient's profession, warrants ongoing monitoring and management, ensuring sustained recovery and preventing potential complications or functional limitations. The application of transcranial Direct Current Stimulation (tDCS) in the management of acute pain and functional recovery following a radial capitellum fracture. Firstly, it represents a shift towards innovative, non-pharmacological pain management strategies, crucial in the current medical landscape where opioid dependency and medication overuse are major concerns. The use of tDCS offers a safer alternative, reducing the risk of medication-related side effects and dependency issues [12]. Secondly, this case demonstrates the potential of tDCS to directly influence neuronal activity within the motor cortex and pain matrix [13]. By modulating these areas, tDCS can effectively alter pain perception and enhance motor recovery, which is particularly crucial in patients requiring rapid and effective return to functional activities, such as those in physiotherapy professions [14]. The significant improvement in pain levels (NPRS from 6 to 0) and functional scores (DASH from 45/100 to 14.2) post-tDCS intervention in our case is a testament to its efficacy. Moreover, this approach could change clinical practice by introducing an effective, non-invasive, and patient-friendly option in pain management protocols, especially in orthopedic settings. It underscores the need for an interdisciplinary approach, combining orthopedic, pain management, and rehabilitation expertise, to optimize patient outcomes. This is particularly relevant in managing fractures like the MASON 2 radial capitellum, where a balance between immobilization for stability and mobilization for preventing stiffness is critical. However, it is important to acknowledge the variability in individual responses to tDCS and the need for research into optimal dosages and electrode placements [15]. Developing standardized guidelines for tDCS application in diverse clinical scenarios will be a significant step forward in this field.

## 5 Conclusion

The application of tDCS in this context is not only important for its immediate benefits in pain reduction and functional improvement but also for its potential to change clinical practice by providing a safe, effective, and patient-centered alternative to traditional pain management strategies. This case highlights the need for further research into the mechanisms, efficacy, and standardization of tDCS, paving the way for its broader application in clinical settings and ultimately enhancing patient care and outcomes.

## Ethical approval

Ethical approval is not a requirement at our institution for reporting individual cases or case series.

## Funding

Authors state no funding involved.

## CRediT authorship contribution statement

RT contributed to conception and design of the study; RT to data acquisition, RT and DP to data analysis and interpretation; RT and DP contributed to draft the manuscript; RT and LBcontributed to the critical revision for important intellectual content. All authors read and approved the final version of the manuscript.

## Guarantor

Roberto Tedeschi.

## Informed consent

Consent in format was obtained from the patient.

## Declaration of competing interest

Authors state no conflict of interest.
